# Applied Electric Fields Polarize Initiation and Growth of Endothelial Sprouts

**DOI:** 10.1155/2023/6331148

**Published:** 2023-12-23

**Authors:** Anyesha Sarkar, Shanta M. Messerli, Md Moin Uddin Talukder, Mark A. Messerli

**Affiliations:** ^1^Department of Biology and Microbiology, South Dakota State University, Brookings, SD 57007, USA; ^2^Sanford Research, Sioux Falls, SD 57104, USA; ^3^Department of Biomedical Engineering, University of South Dakota, Sioux Falls, SD 57107, USA

## Abstract

Therapeutic electric fields (EFs) are applied to the epidermis to accelerate the healing of chronic epidermal wounds and promote skin transplantation. While research has emphasized understanding the role of EFs in polarizing the migration of superficial epidermal cells, there are no reports describing the effect of EFs on polarization of the underlying vasculature. We explored the effects of EFs on the growth of endothelial sprouts, precursors to functional blood vessels. We discovered that DC EFs of the same magnitude near wounded epidermis polarize initiation, growth, and turning of endothelial sprouts toward the anode. While EFs polarize sprouts, they do not change the frequency of primary sprout or branch formation. Unidirectional electrical pulses also polarize sprouts based on their time-averaged EF magnitude. Sprout polarization occurs antiparallel to the direction of electrically driven water flow (electro-osmosis) and is consistent with the direction of sprout polarization induced by pressure-driven flow. These results support the role of EFs in controlling the direction of neovascularization during the healing of soft tissues and tissue engineering.

## 1. Introduction

Mass transfer through the cardiovascular system is required for cell function and survival in most tissues and organs. Therefore, rebuilding of the vasculature through neovascularization is required for successful tissue repair, reconstruction, and replacement [1, 2]. Neovascularization involves the formation of endothelial sprouts, precursors to mature blood vessels [3]. Endothelial cell migration and sprout growth are guided into the necessary regions of tissues and organs through a combination of chemotactic, haptotactic, and mechanotactic cues [4, 5]. Controlling the growth direction of endothelial sprouts would be most useful to promote *in vivo* tissue repair and construction of vascularized replacement tissues and organs.

We tested the hypothesis that sprout growth may also be polarized by electrical cues. *In vitro*, applied electric fields (EFs) below the sensory threshold of electrically excitable cells polarize cells and direct their migration (galvanotaxis) and growth (galvanotropism) [6–8]. It has been proposed that applied EFs polarize cells by concentrating cell surface receptors toward one of the electrical poles [9, 10]. However, EFs also promote electrokinetic perfusion, i.e., electrically driven water flow (electro-osmosis) in the interstitial space [11, 12], and could generate mechanical signals that polarize cells.

*In vitro*, EFs polarize and direct the migration of endothelial progenitor cells and endothelial cells toward one of the electrical poles [13–15]. *In vivo*, electrical stimulation enhances the growth of the local vasculature [16, 17]. However, nothing is reported about the electrical sensitivity of the direction of endothelial sprout growth. *In vitro*, endothelial sprouts develop from sheets or clusters of endothelial cells [18, 19]. Here, we report our discovery that EFs polarize the initiation, growth, and turning of human umbilical vein endothelial cell (HUVEC) sprouts toward the anode, the opposite direction of migration of individual HUVECs under similar conditions. We explore DC, pulsed, and alternating EFs to test the mechanisms by which the nonexcitable sprouts sense EFs. We also provide *in vitro* evidence that transepidermal electrical stimulation could promote sprout polarization *in vivo*.

## 2. Materials and Methods

Studies of endothelial sprout growth in fibrin gels were performed using cultures of endothelial sheets on Cytodex 3 microcarrier beads (Sigma-Aldrich, St. Louis, MO) and cultures of endothelial cell clusters. Endothelial cell structures were grown in coculture with skin fibroblasts to promote sprout formation and exposed to EFs of 50, 155, and 360 mV/mm in IBIDI channel slides.

### 2.1. HUVEC Cultures

Human umbilical vein endothelial cells (HUVECs, Lonza, Walkersville, MD) were cultured in EGM-2 media consisting of EBM-2 basal media with EGM-2 SingleQuot supplements according to the manufacturer's instructions (Lonza, Walkersville, MD). Endothelial sheets or clusters were formed by culturing cells in 2% agarose wells (Fisher Scientific, Waltham, MA) formed using MicroTissues 3D Petri Dish molds (small spheroids, Sigma-Aldrich, St. Louis MO). After formation in sterile PBS, agarose wells were equilibrated with EGM-2 media prior to adding cells.

Cytodex beads were hydrated and sterilized according to the manufacturer's instructions. Sterile beads were diluted by a factor of 16 in EGM-2 media before they were added dropwise to the agarose wells and allowed to settle. Excess media was removed, and more beads were added until most wells were filled with at least one bead. HUVECs were trypsinized, rinsed in EGM-2 media, and concentrated before they were added dropwise on top of the Cytodex beads in the agarose wells. HUVECs were allowed to form a cell sheet on the beads in a 37°C, 5% CO_2_ incubator for 2.5 days. EGM-2 media was replaced every 24 hrs during endothelial sheet formation.

Agarose wells were also used to culture HUVEC clusters. Cells were added dropwise on top of the molds in EGM-2 media and incubated in the 37°C, 5% CO_2_ incubator for 1.5 days to allow them to form cell spheroids. EGM-2 was replaced every 24 h. Endothelial cell-coated beads or clusters were collected by emptying the agarose wells into EGM-2 in a small, sterile Petri dish and tilting the Petri dish to concentrate the HUVEC structures at the lower edge.

### 2.2. HUVEC Sprout Culture

HUVEC structures were grown in coculture with fibroblasts in fibrin gels to promote sprout formation and eliminate the complications associated with Matrigel [20]. Bovine fibrinogen (20 mg, Alfa Aesar, Ward Hill, MA) was dissolved in 5 mL of filter-sterilized saline (0.9% NaCl, 25 mM HEPES, pH 7.4) overnight and concentrated to 20 mg/mL using a 30 kDa MWCO Vivaspin 6 centrifugal concentrator (GE Healthcare UK Limited, Little Chalfont Buckinghamshire). Bovine thrombin (EMD Millipore Corp., Darmstadt, Germany) was dissolved in filter-sterilized modified Ringer's solution (in mM 145 NaCl, 3 KCl, 2 CaCl_2_, 25 HEPES, pH 7.4) containing 0.1% bovine serum albumin to make a 200 U/mL stock and was diluted to a 50 U/mL working stock using calcium-containing saline (0.9% NaCl, 2.5 mM CaCl_2_, 25 mM HEPES, pH 7.4). Primary epidermal fibroblasts [21] were cultured in phenol-red-free Dulbecco's modified Eagle's medium (DMEM) with 10% FBS and 1% Pen-Strep. Fibroblasts were trypsinized, rinsed, and suspended in 3 mL of EGM-2 medium prior to mixing with HUVEC structures. During endothelial sprout assays, HUVEC-coated beads or HUVEC clusters and fibroblasts were added in a 1 : 10 ratio by volume of suspension media. The final mixture included fibrinogen (5 mg/mL), thrombin (0.4 U/mL), fibroblasts, and HUVEC-coated beads or HUVEC clusters in 1 mL. The culture mixture was poured into IBIDI channel slides (IBIDI *μ*-slide VI 0.4, Fitchburg, Wisconsin) and allowed to gel for 2 min at room temperature, followed by 30 min in a 37°C, 5% CO_2_ incubator. Chambers were then arranged for long-term experiments.

### 2.3. Electric Field Application

**Figure 1:**
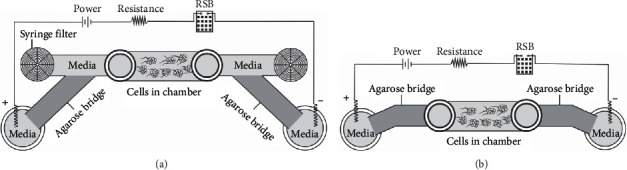
Experimental channel design. EFs were applied to HUVEC structures in IBIDI channel slides. A series resistor and resistance substitution box (RSB) were used to monitor and control the electric field in the channels. (a) The open channel design enabled easier exchange of media by simply removing the syringe filters. This design also provided a low-resistance pathway for fluid flow from the anode to the cathode side of the channel. (b) The closed channel design involved directly connecting the low electroendosmosis (EEO) agarose bridges to the IBIDI wells. While cathode-directed electro-osmotic flow (EOF) continues along negatively charged surfaces through the channel, fluid pressure gradients dissipate in the reverse direction through the porous fibrin matrix.

EFs were applied to experimental chambers after fibrin gels formed. The experimental arrangements of the IBIDI channels are illustrated in Figure 1. DC EFs of 50, 155, and 360 mV/mm were applied to HUVEC structures and sprouts using 9 V battery banks. These field strengths are 4 orders of magnitude lower than the pulsed fields used to disrupt blood supply to superficial melanoma [22]. Pulsed EFs were applied using an arbitrary function generator (AFG 3021, Tektronix, Inc., Portland, OR) controlling a voltage amplifier (Model MDT693, Thorlabs Inc., Newton, NJ) for the 14.3% and 50% duty cycles or controlling a WMA-02 HV amplifier (Falco Systems, Katwijk, Netherlands) for the alternating polarity waveform. Power supplies were connected to IBIDI channel slides as described previously [11]. Pulsed EFs of 360 mV/mm were applied with duty cycles of 14.3% (1 min on and 6 min off) and 50% (1 min on and 1 min off) to maintain time-averaged EFs of 51.4 and 180 mV/mm, respectively. An EF with symmetrical alternating polarity possessed a 2 min period and a half-wave amplitude of 360 mV/mm. In the absence of EFs, channels were connected to Luer tapered L-connectors containing ∼650 *μ*L of medium, greater than 21 times the volume of the cell-loaded channel, on each end of the channel. Chambers were kept in a 37°C, 5% CO_2_ incubator for the duration of the experiment, and media was replaced on each side of the channels and in the Ag/AgCl electrode baths every 12 hours.

Phase-contrast images of sprouts grown in the absence or presence of EFs were collected after 48 h using a Thorlabs camera (Newton, NJ) attached to an Axiovert 40 CFL inverted microscope. Considering the density and growth rate of cultured sprouts, 48 h allowed substantial growth without excessive overlap or anastomosis that would have made sprout analysis more difficult. Primary fibroblasts display delayed and relatively weak polarization in response to EFs [23]. Their migration rates of 10–13 *μ*m/h in the presence of applied EFs [23] indicate that only 0.5–0.6 mm of the ends of the 17 mm long IBIDI channel may have experienced a change in fibroblast density during the 48 h experiment.

Sprouts and their branches were traced using the line segment tool in ImageJ [24]. The ImageJ ROI manager was used to export line segment coordinates for further analysis. MATLAB scripts (MathWorks, Natick, MA) were used to process traced line segments, form trajectory plots, and generate polar plots of sprout characteristics. Sprout initiation is based on the angle of the first traced line segment growing away from the cell cluster, with respect to the *x*-axis. Polar plots were organized into 12, 30° regions. Turning was assessed by calculating the difference in cos*θ*, i.e., Δcos*θ*, between the first and the last sprout segments and plotted in the region of sprout initiation on the polar plots. Cos*θ* is the projection of the sprout segments onto the *y*-axis, e.g., (*y*_1_−*y*_0_)/sqrt((*y*_1_−*y*_0_)^2^ + (*x*_1_−*x*_0_)^2^). The average Δcos*θ* for sprouts exposed to EFs was normalized to Δcos*θ* for sprouts grown in the absence of EFs so that values <1 indicate cathodal turning, a value near 1 indicates no net turning, and values >1 indicate anodal turning.

### 2.4. Statistical Analysis

The statistical significance of polarized sprout initiation, growth, and turning was determined using one-tailed *t*-tests. Statistical significance between other results was compared using one-tailed or two-tailed *t*-tests as listed in the text.

### 2.5. HUVEC 2D Migration

IBIDI channel slides were filled with EGM-2 media for 30 min prior to plating separated HUVECs. Cells were allowed to attach to the channels for 20 min in a 37°C, 5% CO_2_ incubator before starting time-lapse imaging. Cells were kept at 37°C during experimentation. Phase-contrast images were collected in 5 min intervals for 4 h using a Thorlabs camera mounted on a Zeiss Axiovert 25 inverted microscope. Images were first collected for 30 min in the absence of EFs before EFs of 155 and 360 mV/mm were applied as described above. ImageJ was used to trace the periphery of each cell in every frame to calculate the centroid coordinates. Movement of the cell centroid was used to calculate the migration trajectories of the individual HUVECs. Cos*θ* was used, as described above, to assess polarized migration toward the electrical poles with cathodal migration designated as positive.

### 2.6. EOF through Fibrin Gel

Electro-osmotic flow (EOF) through fibrin gels was measured at ambient temperature in EGM-2 media by tracking the dye front of neutral Texas red dextran (3 kDa, ex./em. 595/615 nm, Invitrogen, Waltham, MA). Time-lapse images of the dye front were acquired at 15 s intervals in the absence and presence of 1000 mV/mm DC EFs (Lambda Electronics Inc., Melville, N.Y.) using an experimental setup and analysis as described previously [11].

## 3. Results

**Figure 2:**
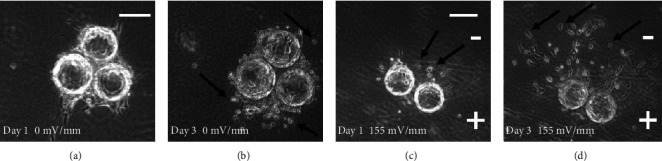
Photos of endothelial cell migration in fibrin gels in the absence of fibroblasts. (a) After one day in fibrin gel, the endothelial cells remained tightly adhered to the collagen-coated beads and small sprouts formed. (b) After three days in the absence of fibroblasts, endothelial cells had detached from the beads and migrated into the fibrin gel (black arrows). (c) In the presence of a 155 mV/mm DC EF, some cells started migrating toward the cathode in the fibrin gel (black arrows) after one day. (d) After 3 days in the EF, many of the cells had detached from the beads and migrated toward the cathode pole in the fibrin gel (black arrows) at significantly greater distances than in the absence of the EF shown in Figure 2(b) (scale bars 100 *μ*m).

**Figure 3:**
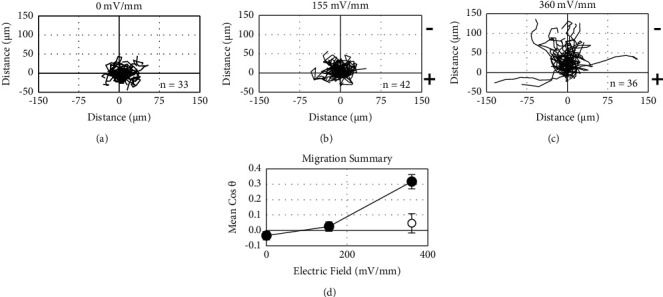
EFs direct single endothelial cell migration toward the cathode. (a–c) Trajectory plots for single migrating HUVECs in the absence and presence of applied DC EFs over a period of 4 hours. (d) HUVECs cultured on glass migrate toward the cathode in an applied EF (●); cathode is positive. When exposed to 360 mV/mm, HUVECs migrate randomly on tissue culture plastic (○) (*n* = 11).

In the absence of fibroblasts, sheets and clusters of HUVECs dissociate in fibrin gels containing EGM-2 media (Figures 2(a) and 2(b)). Under those conditions, individual HUVECs migrate toward the cathode (negative pole) in DC EFs (Figures 2(c) and 2(d)) similar to their migration direction in 2D culture (Figure 3).HUVEC sheets on the large Cytodex beads were considered to distort the local EF, so most experimentation with sprout polarization occurred with the smaller HUVEC clusters.

**Figure 4:**
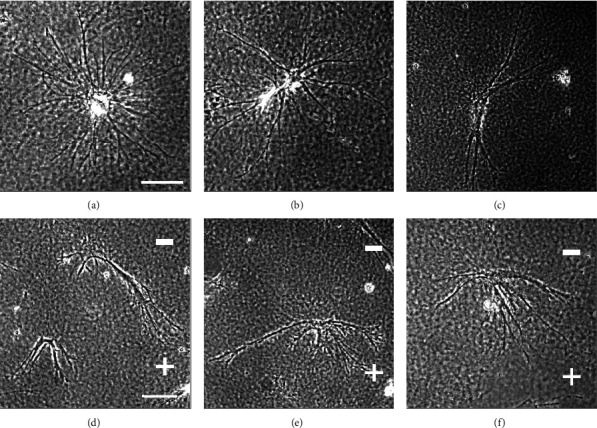
Photos of endothelial sprouts. (a–c) Sprouts grow persistently away from the cell mass in the absence of applied EFs. (d–f) Sprouts grow toward the anode in the presence of DC EFs of 360 mV/mm (scale bars 100 *μ*m).

**Figure 5:**
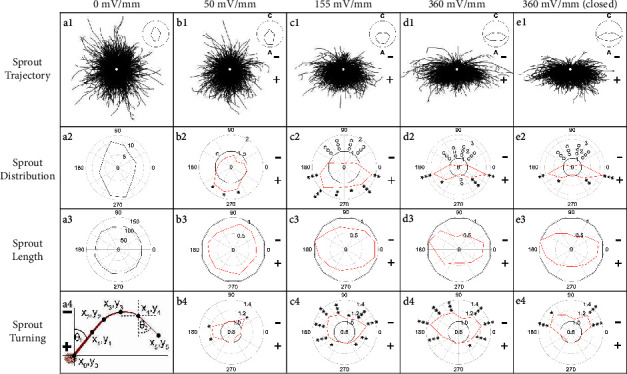
DC EFs polarize endothelial sprouts. (a1–e1) Superimposed sprout tracings display increasing anodal polarization with EF strength (origin = white disk). Insets display the relative distribution of all sprouts for each 30° region of each condition. C: cathode; A: anode. (a2) Regional comparison of relative sprout distribution in the absence of EFs (100 • Σ sprout length per region/Σ sprout length for all regions). (b2–e2) Relative distribution of EF-exposed sprouts (red) normalized to the relative sprout distribution determined in the absence of EFs (black) for each condition. (a3) The average sprout length (*μ*m) remains the same for all regions in the absence of EFs. (b3–e3) EFs decrease average relative sprout length (red) compared to sprouts grown simultaneously in the absence of EFs (black). (a4) Sprouts were traced using the line segment tool in ImageJ and used to determine the Δ cos *θ* values between the initial segment (*θ*_1_) and the final segment (*θ*_2_) of each sprout. (b4–e4) Relative average turning of EF-exposed sprouts (red) normalized to sprout turning determined in the absence of EFs (black). Cathodal turning (<1), no net turning (=1), anodal turning (>1). One-tailed *t*-tests were used to assess statistical significance for increased (^*∗*^) or decreased (∘) growth or turning (one symbol, 0.002 < *p* < 0.02; two symbols, 0.0002 < *p* < 0.002; three symbols, *p* < 0.0002).

In coculture with skin fibroblasts, HUVEC clusters remain stationary and initiate sprouts that grow persistently away from the HUVEC mass (Figures 4(a)–4(c)). Fibroblasts provide a number of factors that promote sprout formation in the absence of EFs [25] and are expected to be necessary in the presence of EFs. Sprouts and their branches were traced for further analysis (Figure 5 a1–e1). In the presence of EFs, cell sheets on collagen-coated beads remain stationary and display sprout growth toward the anode, indicating that no movement of the central cell mass is required for the asymmetry in sprout growth.

In the absence of EFs, the asymmetry between vertical and horizontal growing sprouts (Figure 5a1-a2) reflects the long (17 mm) and short (3.8 mm) axes of the IBIDI culture channels, respectively, and the fact that sprouts that grew against the channel side walls were not quantitated. Therefore, the growth of EF-exposed sprouts was compared to the growth of sprouts in the absence of EFs for simultaneous cultures.

### 3.1. Sprout Initiation

Sprout growth is polarized toward the anode (positive pole) at 50 mV/mm and increases with field strength (Figures 4(d)–4(f) and 5 b1-d1). The relative distribution of EF-exposed sprouts (Figure 5 b2-d2) (red) indicates significantly greater anodal growth and lesser cathodal growth compared to sprouts grown in the absence of EFs (black). EFs did not significantly alter the average number of primary sprouts per channel (Table 1).

**Figure 6:**
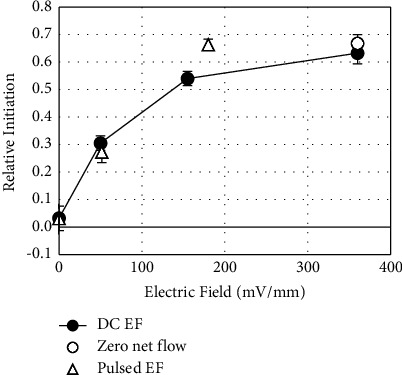
EFs direct sprout initiation toward the anode. (●) DC EFs significantly polarize sprout initiation toward the anode at 50 mV/mm and increase anodal initiation at 155 and 360 mV/mm. (○) Polarized initiation toward the anode at 360 mV/mm in the closed channels, where there is no net fluid flow, is not statistically different than initiation in the open channels at the same field strength (*p* > 0.5). (△) Pulsed EFs of 360 mV/mm with alternating direction, possessing a time-averaged EF of 0 mV/mm, showed no difference in the direction of initiation. Unidirectional pulsed EFs of 360 mV/mm with 14.3% and 50% duty cycles with time-averaged EFs of 51.4 and 180 mV/mm, respectively, significantly polarize sprout initiation toward the anode (*p* < 0.01 and *p* < 0.0001).

Increased anodal growth is accompanied by polarization of primary sprout initiation (Figure 6). In the absence of EFs, sprout initiation is evenly distributed across the *x*-axis, with a relative initiation direction close to zero, 0.03 ± 0.02. In the presence of EFs, the direction of initiation ((Σ number of anode-initiating sprouts–Σ number of cathode-initiating sprouts)/Σ total number of sprouts) is significantly polarized toward the anode at 50 mV/mm, 0.31 ± 0.03 (*p* < 0.002 one-tailed *t*-test) and increases to 0.54 ± 0.03 and 0.63 ± 0.04 at 155 and 360 mV/mm, respectively.

### 3.2. Sprout Growth

EFs direct greater relative growth of sprouts and their branches toward the anode and away from the cathode (Figure 5b2-d2). On average, 25.9 ± 5.3% of primary sprouts had branches in the absence of EFs, with no consistent increase in the number of branches in the presence of EFs (*p* > 0.1 for 50 mV/mm (28.1 ± 4.6%) and 360 mV/mm (35.3 ± 4.1%), *p* < 0.02 for 155 mV/mm (36.4 ± 11.3%), two-tailed *t*-test). In the absence of EFs, the relative initiation direction for branches is 0.10 ± 0.12. EFs significantly polarize branch initiation toward the anode, i.e., 0.53 ± 0.14 at 50 mV/mm (*p* < 0.005) and 0.65 ± 0.05 and 0.67 ± 0.07 at 155 and 360 mV/mm, respectively (*p* < 10^−5^ for both conditions).

Exposure to 50 mV/mm significantly reduced relative growth toward the cathode at 30–60° and 120–150° and increased relative sprout growth toward the anode at 210–300° (Figure 5 b2). On average, larger EFs decreased the relative cathodal growth of cathode-initiating sprouts at 30–150° and increased the relative anodal growth of anode-initiating sprouts at 180–360° (Figure 5 c2-d2). At 155 and 360 mV/mm, relative lateral-anodal sprout growth at 180–240° and 300–360° is significantly greater than growth directly facing the anode at 240–300° (*p* < 10^−3^ for both conditions).

While anodal polarization increases, the average sprout length decreases with increasing field strength. The average sprout length after 48 h in the absence of EFs is 112.0 ± 2.4 *μ*m, ranging between 108.3 and 115.8 *μ*m for each of the 12 polar regions. When exposed to 50, 155, and 360 mV/mm, the average sprout length decreased to 0.75 ± 0.05 (range 0.67–0.83), 0.79 ± 0.12 (range 0.63–1.01), and 0.62 ± 0.18 (range 0.41–0.97) (mean ± s.d.) of sprouts grown in the absence of EFs, respectively (Figure 5 b3-d3). In EFs of 155 and 360 mV/mm, sprout length parallel to the EFs (60–120° and 240–300°) is reduced to 0.67 and 0.44 of sprout length in the absence of EFs, significantly lower than 0.93 and 0.79 (*p* < 10^−8^ for both comparisons, one-tailed *t*-test), the average relative sprout lengths perpendicular to the EFs (330−30° and 150–210°).

### 3.3. Sprout Turning

EFs also enhance turning of primary sprouts toward the anode. Average turning was assessed by calculating the average Δcos*θ* between the initial and final line segments used to trace each primary sprout, illustrated in Figure 5 a4. Each region of the polar plots in Figure 5b4-d4 indicates the average Δcos*θ* for the sprouts that initiated in that region. In the absence of EFs, the average Δcos*θ* for the 12 different regions is 1.01 ± 0.06, indicating no net turning trend. Exposure to 50 mV/mm (Figure 5 b4) significantly increases turning of sprouts toward the anode at 30–60° and 150–210°. In the presence of 155 and 360 mV/mm (Figure 5c4-d45), anodal turning increases for most of the regions outside 240–300°. Sprouts that initiate toward the anode, 240–300°, grow persistently toward the anode. Sprouts that initiate at angles perpendicular to the EF or toward the cathode show greater average turning away from the cathode.

### 3.4. Role of Extracellular Water Flow

Applied EFs may polarize endothelial sprouts by promoting cathode-directed water flow through the interstitial space [10, 12, 26]. Pressure-driven flow velocity <1 *μ*m/s is sufficient to polarize sprout initiation and growth through collagen and fibrin gels antiparallel to the flow direction [27, 28]. We measured an average electro-osmotic mobility (EOM) of 4.2 ± 0.3 nm/s/V/m through the negatively charged, porous fibrin gels in these experiments, leading to flow velocities of 0.2, 0.7, and 1.5 *μ*m/s in applied EFs of 50, 155, and 360 mV/mm, respectively.

**Figure 7:**
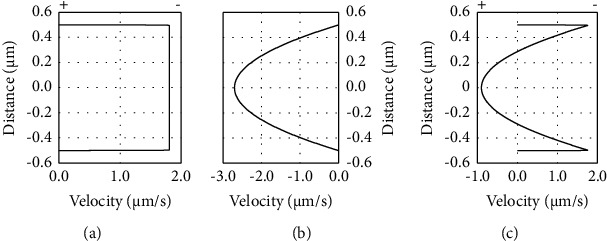
Cathode-directed electro-osmotic flow continues near negatively charged surfaces during zero net fluid flow in a closed channel. (a) Electro-osmotic flow toward the cathode is modelled through a 1 *μ*m tall channel forming a plug flow profile [29]. (b) A reverse direction pressure gradient is modelled and generates a parabolic flow profile through the 1 *μ*m tall channel with the same average volumetric flow rate. (c) Simultaneous application of both flows generates slightly reduced flow velocity next to the charged walls, and backflow through the lowest resistance pathway, the center of the channel. *In vitro*, fibrin gels have pore sizes ranging from 3 to 10 *μ*m depending on preparation conditions [30, 31]. Therefore, EOF next to charged fibrin and charged sprout surfaces continues in a closed system and the pressure gradients dissipate through the low resistance center of the pores.

The initial experiments used for studying polarization of sprouts were performed in “open” channels (Figure 1(a)) to enable media exchange. The arrangement enabled unidirectional electro-osmotic flow (EOF) of media through the culture channels [11, 12]. To mimic endogenous conditions, sprouts were also exposed to EFs in “closed” channels (Figure 1(b)) where fluid pressure gradients would dissipate via backflow through the center of the low-resistance fibrin pores, resulting in zero net fluid flow through the channel (Figure 7).

In the closed channels, cathode-directed EOF continues against negatively charged surfaces [32] including fibrin extracellular matrix and endothelial cell membranes, i.e., we predict that the EOM at the surface of endothelial cells is 0.7 nm/s/V/m based on the zeta potential of endothelial cells [33]. Under the conditions of no net fluid flow, sprout initiation, growth, and turning are polarized toward the anode (Figure 5e1-e4 and Figure 6), and there is no significant change in the number of primary sprouts (Table 1) (*p* > 0.5 two-tailed *t*-test).

In the absence of net fluid flow in the closed channels, relative sprout length facing the anode (Figure 5 e3) is significantly longer than in the same region exposed to unidirectional flow in the open channels (Figure 5 d3), 240–300° (*p* < 0.005) or 210–330° (*p* < 0.02). No significant difference exists in relative sprout length between these two conditions in the regions facing the cathode between 60 and 120° (*p* > 0.5) or 30–150° (*p* > 0.5). Also, no significant anodal turning occurs in closed channels on the anode side between 180 and 360° (Figure 5 e4). These results support a role for fluid flow during the reduction of anodal growth length and during anodal turning. They also support the hypothesis that application of DC EFs *in vivo* could polarize sprout initiation and growth in porous spaces beneath the skin.

### 3.5. Asymmetric Pulsed EFs Polarize Sprouts

**Figure 8:**
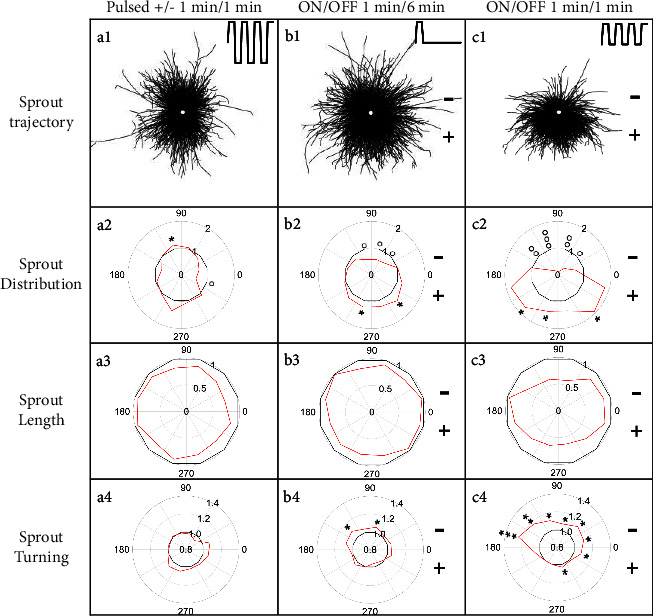
Sprout polarization is dependent on the time-averaged field strength. (a1–c1) Unidirectional pulsed EFs increase sprout polarization. Insets show the applied electrical waveforms beginning at 0 mV/mm. (a2–c2) Relative sprout distribution exposed to pulsed EFs (red) compared to the normalized sprout distribution determined in the absence of EFs (black). (a3–c3) Relative length of sprouts exposed to pulsed EFs (red) compared to normalized sprout length in the absence of EFs (black). (a4–c4) Sprout turning toward the anode, Δ cos *θ* > 1, increases with time-averaged EF. One-tailed *t*-tests were used to assess statistical significance for increased (^*∗*^) or decreased (∘) growth or turning (one symbol, 0.002 < *p* < 0.02; two symbols, 0.0002 < *p* < 0.002; three symbols, *p* < 0.0002).

To explore asymmetries in response time between polarized anodal growth and growth inhibition toward the cathode, EFs of 360 mV/mm were alternated each minute, creating a time-averaged EF of zero. Under these conditions, sprouts are not polarized (Figure 8 a1). There is no significant polarization of sprout initiation between the two poles (Figure 6) or even between the more restricted regions where sprout initiation is parallel to the EFs between 60–120° and 240–300° (*p* > 0.1, two-tailed *t*-test). There is no significant difference in the number of primary sprouts in the absence of EFs or the presence of alternating EFs (Table 1) (*p* > 0.1). No consistent differences were identified for sprout distribution in the regions encompassing 60–120° or 240–300° (Figure 8 a2) (*p* > 0.1), but the average sprout length was significantly reduced to 0.86 ± 0.07 of sprouts not exposed to EFs (Figure 8 a3) (*p* < 0.001). No significant turning was identified compared to sprouts grown in the absence of EFs (Figure 8 a4).

Further experiments were performed with unidirectional, pulsed EFs using 360 mV/mm with duty cycles of 14.3% and 50%, leading to time-averaged EFs of 51.4 and 180 mV/mm, respectively (Figure 8 b1-c1). Under these conditions, sprout initiation (Figure 6) and growth (Figure 8 b2-c2) are significantly polarized to the anode. There was no significant difference between the number of primary sprouts in the absence of EFs or presence of the pulsed EFs at 14.3% duty cycle or 50% duty cycle (Table 1) (*p* > 0.1 and *p* > 0.5, respectively). Relative sprout length in the presence of pulsed EFs (14.3% duty cycle) (Figure 8 b3), 0.89 ± 0.06, is significantly greater than the relative sprout length in DC EFs of 50 mV/mm, 0.75 ± 0.5 (*p* < 10^−5^, one-tailed *t*-test). Significant anodal turning also occurs at two cathode facing regions under these pulsed conditions (Figure 8 b4). Sprouts exposed to 360 mV/mm (50% duty cycle) have an average sprout length of 0.76 ± 0.13 of control sprouts (Figure 8 c3). The average relative sprout length parallel to the EFs, 0.62 ± 0.06, is significantly shorter than the average relative length perpendicular to the EFs, 0.90 ± 0.08 (*p* < 10^−8^). Significant anodal turning occurs for all cathode-initiating sprouts and for two regions of the anode-initiating sprouts (Figure 8 c4). Unidirectional pulsed EFs appear just as efficient at polarizing sprout growth as DC EFs but enable the growth of longer sprouts.

## 4. Discussion

Electrical therapies consisting of DC and pulsed EFs accelerate the healing of chronic epidermal wounds and promote skin transplantation [34–37]. They are thought to work by mimicking or enhancing the native electric fields that occur near the wounded epidermis (100–200 mV/mm) and are capable of polarizing cell migration and growth [6, 38]. These injury-induced EFs expose local cells and extracellular matrix to a polarizing cue until the epidermal barrier and electrical resistance reform.

We provide the first evidence that EFs of similar magnitude as those near epidermal wounds direct HUVEC migration toward the cathode (Figures 2 and 3) and polarize endothelial sprout initiation, growth, and turning toward the anode (Figures 4–6 and 8). Sprout growth, like endothelial cell migration, appears to be dependent on guidance by one cell. The sprout tip cell is hypothesized to migrate and pull on the basal stalk cells prompting them to proliferate and increase the length of the sprout behind the tip cell [39]. This programmed reversal of electrically induced polarity is not unique. *In vitro*, neural crest cells migrate toward the cathode and may respond to endogenous electrical gradients in the embryo to direct their migration [40]. However, after neural crest cells differentiate into Schwann cells, their migration reverses to the anode [41]. These developmentally programmed reversals of EF-induced polarity may be important for cells to make use of steady endogenous EFs as guidance cues during development and repair [6, 42].

We hypothesize that electrically driven water flow, electro-osmosis, promotes sprout polarization similar to pressure-driven flow [27, 28, 43]. Sprout growth in extracellular matrices is antiparallel to pressure-driven perfusion and antiparallel to electrokinetic perfusion. Pressure-driven flow velocities of only 0.3–1.0 *μ*m/s polarize sprout initiation and growth [27, 28] and are similar to the electro-osmotic flow velocities, 0.2–1.5 *μ*m/s, identified in these experiments. *In vitro*, low flow velocities induced by pressure-driven flow eliminate extracellular morphogen gradients and still polarize sprouts antiparallel to flow, supporting mechanotransduction as the polarizing mechanism in response to flow [43].

Unidirectional EFs stimulate anodal initiation and inhibit cathodal initiation (Figure 6), leading to 0.9-, 2.3-, and 4.1-fold greater anodal initiation for 50, 155, and 360 mV/mm, respectively, and 0.8- and 4.0- fold greater anodal initiation for pulsed EFs with 51.4 mV/mm and 180 mV/mm time-averaged EFs. Pulsed EFs at 51.4 mV/mm were just as effective in polarizing sprout initiation as DC EFs with 50 mV/mm (*p* > 0.5), and pulsed EFs at 180 mV/mm were just as effective as DC EFs at 360 mV/mm (*p* > 0.5).

The electro-osmotic flow surrounding the sprouts is sufficient to polarize sprout initiation and growth in the absence of net flow through the fibrin matrix (Figure 5 e1-e4). In closed channels, sprout initiation was still polarized to the anode to the same extent as sprout initiation in open channels (Figure 6). We conclude that net fluid flow through the fibrin is not required to polarize sprout initiation.

Applied EFs induce endothelial sprouts to turn and grow toward the anode similar to trajectory plots for some migrating cells [44] and growing spinal neurites [6]. Endothelial sprout turning was more common with higher magnitude EFs (Figures 5b4-d4 and 8 b4-c4), but was significantly altered in the absence of net fluid flow (Figure 5 e4), indicating that unidirectional fluid flow was, in part, causing the sprouts to turn into the direction of flow.

EFs significantly reduced average sprout length, compared to sprouts grown in the absence of EFs (Figures 5b3-e3 and 8 a3-c3). DC EFs of 360 mV/mm had the greatest effect in reducing average sprout length. However, the DC EFs of 50 mV/mm had a greater effect in reducing sprout length than the 360 mV/mm pulsed EFs with 14.3% duty cycle that produced a time-averaged EF of 51.4 mV/mm. This indicates that the pulsed EFs are not only efficient at polarizing sprout growth but also promote the growth of longer sprouts. Consistent with this hypothesis, sprout growth toward the anode is significantly more impaired parallel to the EF vector compared to sprout growth perpendicular to the EF vector.

Shorter anode-growing sprouts may be a result of having to grow against fluid flow. The average length of sprouts exposed to alternating EFs was reduced to 86% of the average sprout length in the absence of EFs. There would have been no net movement of extracellular growth factors in alternating EFs; however, alternating fluid flow would be present at a rate of 1.5 *μ*m/s through the fibrin matrix. Also consistent with this hypothesis, sprouts grown in a DC EF of 360 mV/mm in the absence of net fluid flow (Figure 5 e3) grew significantly longer in the region facing the anode 210–330° than sprouts exposed to the same EF strength in the open channels exposed to unidirectional fluid flow toward the cathode (Figure 5 d3). While EFs promoted sprout polarization toward the anode and antiparallel to EOF, the flow rate appears to have impeded anodal growth length, especially at the higher DC EFs.

**Figure 9:**
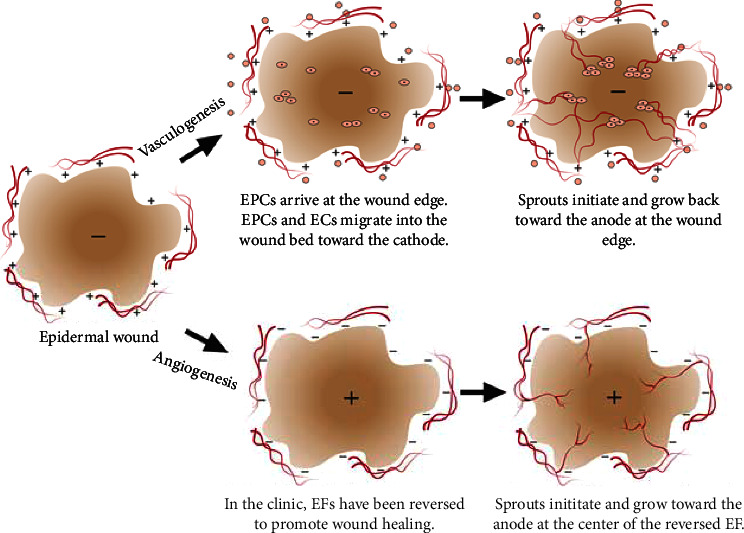
Models describing electrical sprout polarization during normal wound healing (top) and when therapeutic applied EFs are reversed (bottom). (Top) Endothelial precursor cells (EPCs) and endothelial cells (ECs) migrate toward the cathode in applied EFs and are predicted to migrate to the center of a wound based on the native electrical polarity. Initiation and growth of endothelial sprouts originating from the EPCs and ECs near the center of the wound bed will be directed back toward the edge of the wound, the electrical anode. (Bottom) Reversal of the native wound EF has also been found to increase the rate of healing of chronic epidermal wounds. Under these conditions, sprouts at the edge of the wound would initiate and grow toward the center of the wound, promoting vascularization of the wound bed.

We hypothesize that the changes in electrical polarity between migrating endothelial cells and growing endothelial sprouts help to promote revascularization during wound healing. The native electrical polarity of the epidermal wound bed [6] could promote vasculogenesis by directing single endothelial precursors or endothelial cells toward the center of the wound (cathode) (Figure 9) (top). Endothelial sprouts that form in the wound bed after vasculogenesis will be directed back toward the edge of the wound (anode) where they can anastomose with the existing vasculature. Under pathological conditions in chronic wounds that appear to stall in the inflammatory phase of healing [34], applied EFs may promote EOF in the interstitial space. Enhanced interstitial flow may flush inflammatory mediators out of the wound bed while enhancing access to nutrients necessary to support the proliferation and remodeling phases of wound healing [11, 12]. In addition, applied EFs may amplify and extend the native EFs and EOF further away from the wound site to promote revascularization of the wound which is necessary for healing [34].

Reversal of the electrical polarity of epidermal wounds, by placing the anode electrode on the wound bed, is also reported to promote wound healing [35, 45] (Figure 9 (bottom)). Under these conditions, EFs could promote angiogenesis and sprout growth into the wound bed from vessels surrounding the wound. The reversed EFs may be especially necessary to help treat chronic wounds that are associated with poor or damaged vasculature or skin transplants that require vascularization to promote survival.

Electro-osmotic flow has significant advantages over pressure-driven flow in constrained spaces in tissues. EOF makes use of the native negative charge of extracellular matrices and cell surfaces to promote relatively uniform flow velocity between pores of different dimensions [11, 12] while pressure-driven flow is impeded by both the local charge [46] and pore size-dependent drag, according to Poiseuille. As a result, pressure-driven flow causes tissue compaction and increases hydraulic resistance [47, 48]. EOF can be induced across the skin due to electroporation [49], and the flow path can be predicted between two electrical poles based on the electrical resistance in the extracellular space. We hypothesize that EFs applied to promote the healing of chronic epidermal wounds and transplantation of skin can influence the polarization and growth of endothelial sprouts into the wound bed *in vivo* and may be a critical factor in promoting healing. EFs may also be useful to reduce or prevent neovascularization of tissues such as the cornea that can lead to blindness [50].

## Figures and Tables

**Table 1:** Summary of primary sprout number.

Average field strength (mV/mm)	Primary sprout number per channel (mean ± s.d.)
DC control 0	629 ± 271 (*n* = 16)
DC 50	912 ± 291 (*n* = 3)^†^
DC 155	536 ± 270 (*n* = 10)^†††^
DC 360 (open channels)	819 ± 592 (*n* = 6)^†††^
DC 360 (closed channels)	706 ± 234 (*n* = 4)^†††^

Alternating control 0	750 ± 85 (*n* = 3)
Alternating (±360) 0	844 ± 53 (*n* = 3)^††^

Pulsed control 0	959 ± 111 (*n* = 6)
Pulsed 51.4	1206 ± 548 (*n* = 3)^††^
Pulsed 180	1154 ± 115 (*n* = 3)^†††^

(^†^*p* > 0.05, ^††^*p* > 0.1, and ^†††^*p* > 0.5).

## Data Availability

Data used in this study are available upon request to the corresponding author.
